# Synthesis of Polyamidoamine Dendrimer for Encapsulating Tetramethylscutellarein for Potential Bioactivity Enhancement

**DOI:** 10.3390/ijms161125956

**Published:** 2015-11-04

**Authors:** Daniel M. Shadrack, Egid B. Mubofu, Stephen S. Nyandoro

**Affiliations:** 1 Chemistry Department, University of Dar es Salaam, College of Natural and Applied Sciences, P.O. Box 35061 Dar es Salaam, Tanzania; dmssjut@gmail.com; 2 Chemistry Department, St John’s University of Tanzania, P.O. Box 47 Dodoma, Tanzania

**Keywords:** PAMAM G4 dendrimer, encapsulation, tetramethylscutellarein, solubilization, *in vitro* release, stability

## Abstract

The biomedical potential of flavonoids is normally restricted by their low water solubility. However, little has been reported on their encapsulation into polyamidoamine (PAMAM) dendrimers to improve their biomedical applications. Generation four (G4) PAMAM dendrimer containing ethylenediaminetetraacetic acid core with acrylic acid and ethylenediamine as repeating units was synthesized by divergent approach and used to encapsulate a flavonoid tetramethylscutellarein (TMScu, **1**) to study its solubility and *in vitro* release for potential bioactivity enhancement. The as-synthesized dendrimer and the dendrimer–TMScu complex were characterized by spectroscopic and spectrometric techniques. The encapsulation of **1** into dendrimer was achieved by a co-precipitation method with the encapsulation efficiency of 77.8% ± 0.69% and a loading capacity of 6.2% ± 0.06%. A phase solubility diagram indicated an increased water solubility of **1** as a function of dendrimer concentration at pH 4.0 and 7.2. *In vitro* release of **1** from its dendrimer complex indicated high percentage release at pH 4.0. The stability study of the TMScu-dendrimer at 0, 27 and 40 °C showed the formulations to be stable when stored in cool and dark conditions compared to those stored in light and warmer temperatures. Overall, PAMAM dendrimer-G4 is capable of encapsulating **1**, increasing its solubility and thus could enhance its bioactivity.

## 1. Introduction

The development of biodegradable polymeric nanoparticles for effective drug and gene delivery has significantly increased in recent years [1]. Of these, polyamidoamine (PAMAM) dendrimers have been of significant interest due to their unique properties, which provide an endless application in drug delivery. For instance, they are used for gene therapy, drug delivery and magnetic resonance imaging [2]. The use of dendrimers in drug delivery or carrier system to increase therapeutic index, efficacies and solubility in aqueous media is well reported [3,4].

One of the intrinsic properties that limit the clinical applications of the potentially active natural products and their synthetic analogues is poor water solubility [5]. A number of techniques have been used to increase solubility of sparingly water-soluble compounds, among them, being the drug–dendrimer complexation [2]. The ability of PAMAM dendrimers to encapsulate and complex with drugs and other biologically active natural products is based on their unique properties such as monodispersity, large surface area, high degree of molecular uniformity, specific size and shape which are significantly different from their classical linear polymer counterparts [6]. Thus, the drug–dendrimer complexation is currently widely used in pharmaceutical industries as an approach to increase stability, solubility, bioavailability and controlled release of drugs [2,6].

**Figure 1:**
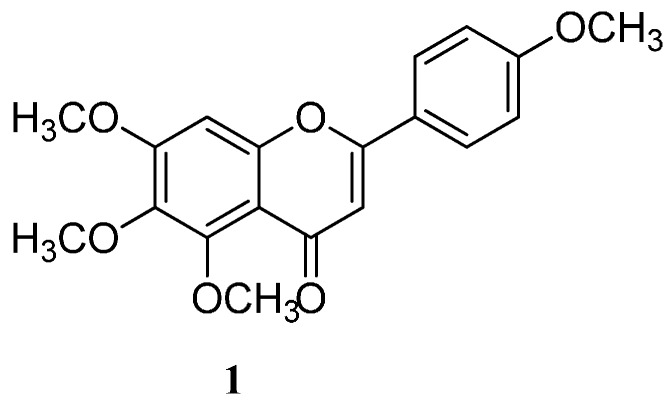
Chemical structure of tetramethylscutellarein (TMScu, **1**).

Flavonoids and other polyphenolic compounds are known to possess cytotoxic activities on different cancer cells and have other biomedical potentials [7,8]. However, water-insolubility and slow dissolution rate remains a serious drawback for their clinical applications [7,9]. Despite the plethora of evidence on PAMAM dendrimers to mediate medical application of most natural products, little have been reported on the encapsulation of flavonoids onto PAMAM and other encapsulating agents [9,10,11]. Thus, the essence of undertaking investigations involving PAMAM and a flavonoid 5,6,7-trimethoxy-2-(4-methoxyphenyl)-4*H*-chromen-4-one (Tetramethylscutellarein (TMScu, **1**), (Figure 1)). The compound is known to exhibit anti-inflammatory properties [12] and moderate cytotoxic activity against human breast cancer cell lines (MCF-7) and anti-tuberculosis potency against *Mycobacterium tuberculosis* (H37Rv strain) [13], whereas its structural analogues are well acknowledged for treatment of various ailments [8,14]. Dendrimers effectiveness for encapsulation, solubolization and drug delivery depends on the number of generations and terminal group among other factors [3,4,5,6]. Hence, our investigations explored the capacity of the amine terminated PAMAM G4 dendrimer to encapsulate compound **1**, a model compound among other bioactive flavonoids [13] for potential bioactivity enhancement. The compound solubilization under the influence of the synthesized G4 PAMAM dendrimer, *in vitro* release and stability in the dendrimer complex were studied, the results of which are reported in this paper.

## 2. Results and Discussion

### 2.1. Synthesis and Characterization of Polyamidoamine (PAMAM) Generation Four (G4) Dendrimer

The synthesis of PAMAM G4 dendrimer was carried out by a divergent approach with reagent excess, wherein ethylenediaminetetraacetic acid (EDTA) was used as core. All synthetic steps were completed and confirmed by copper sulfate color chelation reactions. While half generations gave deep blue color, full generations had purple coloration. The purple color formed was due to the reaction of terminal amine groups present in the dendrimer with copper sulfate, in half generation, the blue color of copper sulfate was observed signifying the absence of terminal amine groups. The Ultraviolet-visible (UV-Vis) spectrometry further provided the proof of synthesis by showing changes in the wavelength values of the dendrimers. The absorption wavelength of the synthesized dendrimer changed from λ_max_ 310 to 299, 291, 287, 279, 264, and 250 nm for G1, G1.5, G2, G2.5, G3, G3.5 and G4, respectively. The change in wavelength was due to the changes in the structure of the dendrimer generations. While the PAMAM dendrimer generation increased, the absorption shifted from high to lower wave numbers.

**Figure 2:**
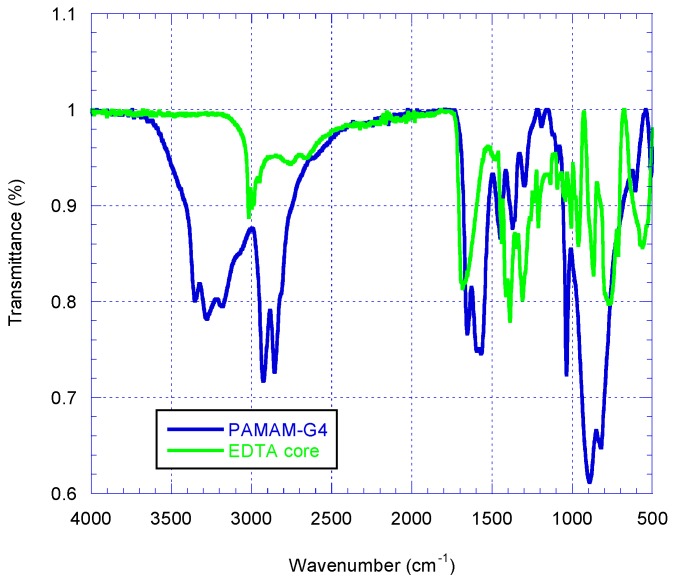
The superimposed IR spectra of solid EDTA (green) and PAMAMG4 (blue).

The Infrared (IR) spectrum of a solid EDTA, a synthetic starting material was acquired parallel with that of the synthesized PAMAM G4 dendrimer for comparison purpose (Figure 2). The IR spectrum of the EDTA showed absorption due to the presence of carboxylic carbon at 1680.9 cm^−1^. The IR spectrum of the synthesized dendrimer G4 showed absorption peaks at 3353.4 cm^−1^ for N–H stretching of primary amine, 3277.4 and 3182.4 cm^−1^ for N–H stretching of secondary amine. The peaks at 2927.9 cm^−1^ and 2856.5 cm^−1^ were for aliphatic C–H stretches. A peak at 1654.5 cm^−1^ was assigned for amide carbonyl absorption while a peak at 1568.4 cm^−1^ was due to a core N–C stretching. A disappearance of the IR absorption band at 1680.9 cm^−1^ for carboxylic carbon for EDTA and appearance of IR absorption at 1654.5 cm^−1^ for the PAMAM G4 dendrimer was a further evidence of the success of the synthetic transformation carried out.

**Figure 3:**
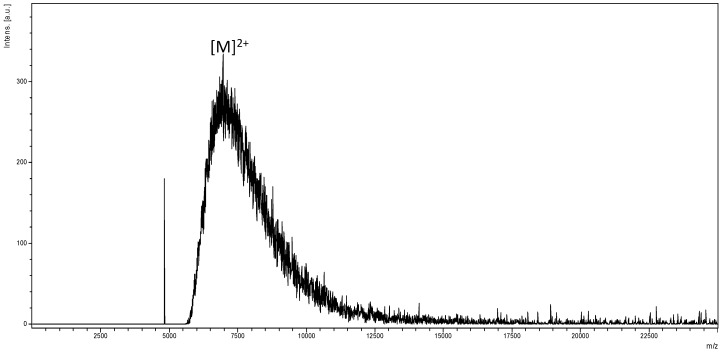
MALDI-TOF mass spectrum of PAMAM G4 acquired using *Trans*-2-(3-(4-*tert*-butylphenyl)-2-methyl-2-propenylidene)malononitrile (DCTB) as a matrix.

The Matrix-Assisted Laser Desorption/Ionization Time of Flight Mass Spectrum (MALDI-TOF MS) shown in Figure 3, does not show appreciable peaks at the region expected for molecular ion corresponding to theoretical Mol. Wt. (14.21 kDa), instead it contains a broad peak at a region ideal for half molecular mass for PAMAM G4 (approximately 7.10 kDa) corresponding to [M]^2+^. Similar phenomenon including molecular mass deviations and dendrimer structural defects under MALDI MS determination has been reported [15,16]. Missing molecular ion and peak broadening at half-size for the desired molecular mass is ascertained to random fragmentations under applied MALDI operating energy, the latter observation coinciding with [M]^2+^ indicates possible presence of as-synthesized PAMAM G4.

**Figure 4:**
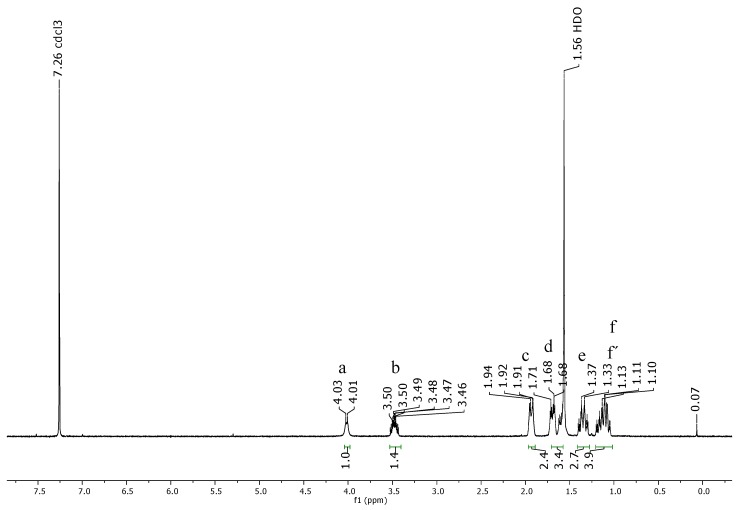
The ^1^H NMR spectrum of PAMAM dendrimer G4 observed at 400 MHz for CDCl_3_ solution at 25 °C.

**Figure 5:**
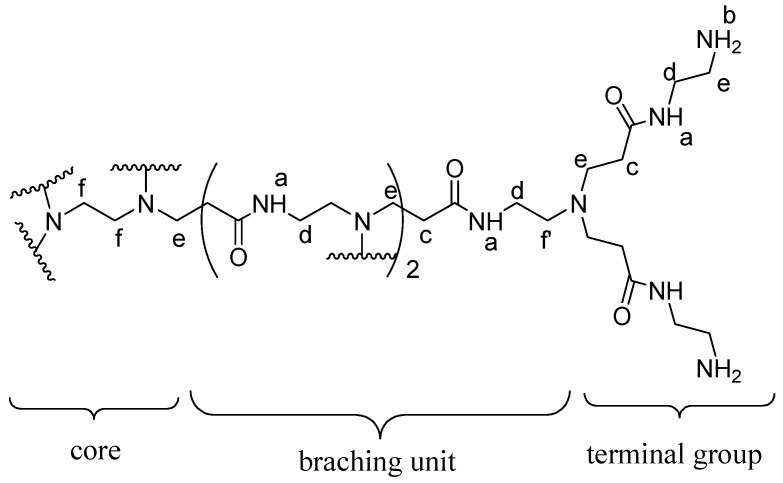
Chemical structure and atom labeling of the part of dendrimer G4.

The proton nuclear magnetic resonance (^1^H NMR) spectrum of PAMAM G4 dendrimer (Figure 4) consisted of set of signals due to only six different protons correlated to partial structure in Figure 5. The proton peaks were designated as H_a_, H_b_, H_c_, H_d_, H_e_ and H_f_. The most deshielded peak resonating at δ 4.02 was assigned to amide protons (H_a_) while the proton signal resonating at δ 3.48 was assigned to terminal amine protons (H_b_). The proton peak resonating at δ 1.93 was assigned to methylene protons near the carbonyl group (H_c_). Furthermore, the peak at δ 1.72 was assigned to methylene protons next to the amido nitrogen (H_d_). An upfield peak resonating at δ 1.69 was assigned to methylene protons (H_e_) adjacent to the terminal amine. The most shielded upfield peak resonating at δ 1.35 was assigned to methylene protons inside the core (H_f_) and those near tertiary amine (H_f′_).

The structural analysis of synthesized dendrimer G4 as represented by atom labeled partial structure (Figure 5) is in agreement with the ^1^H NMR spectrum (Figure 4). The structure consists of six different types of protons. These are amido protons (a), protons at the terminal amino group (b), methylene protons adjacent to carbonyl (c), methylene protons adjacent the amido nitrogen (d), methylene protons next to terminal amino group (e), methylene protons near the tertiary amine (f′) and the methylene protons of the ethylenediaminetetraacetic acid core (f). Generally, results from the UV-Vis, IR, NMR and MALDI-TOF-MS corroborated those reported in previous studies [15,16,17,18,19,20]. Therefore, with these evidences, EDTA core PAMAM G4 dendrimer was successfully synthesized and characterized.

### 2.2. Encapsulation and Characterization of Tetramethylscutellarein (TMScu (**1**))-Dendrimer Complex

**Figure 6:**
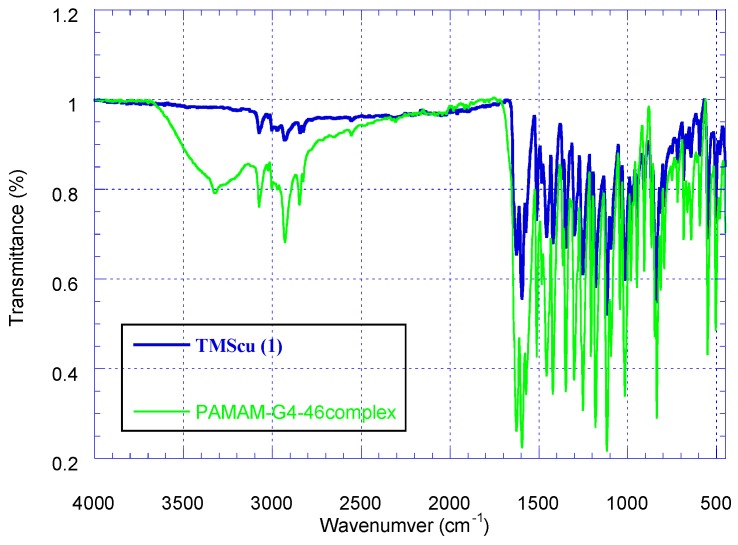
The superimposed IR spectra of TMScu (**1**) and its dendrimer complex.

The encapsulation ability of amine terminated PAMAM G4 dendrimer by complexing with tetramethylscutellarein (TMScu, **1**) was investigated using IR, NMR spectroscopic and *in vitro* release studies. The superimposed IR spectra of TMScu (**1**) and the compound–dendrimer complex are shown in Figure 6. Compound **1** exhibited IR absorption peak at 1626.4 cm^−1^ corresponding to aromatic conjugated carbonyl group. The absorption peak at 3075.1 cm^−1^ was assigned to aromatic ring C–H stretch. In PAMAM G4-**1** complex, a new broad absorption peak appeared between 3500–3100 cm^−1^, which indicated the interaction between the dendrimer and the encapsulated compound. The formation and existence of a broad peak (3500–3100 cm^−1^) with a loop in the region around 3324.1 cm^−1^ in the IR spectra of PAMAM G4-**1** complex suggests the presence of –NH_3_^+^ functionality which results from electrostatic interaction between compound **1** and the dendrimer. Such broadening in IR in the region of NH and NH_2_ due to formation of –NH_3_^+^ is comparable to previously reported encapsulation studies [5,21].

**Figure 7:**
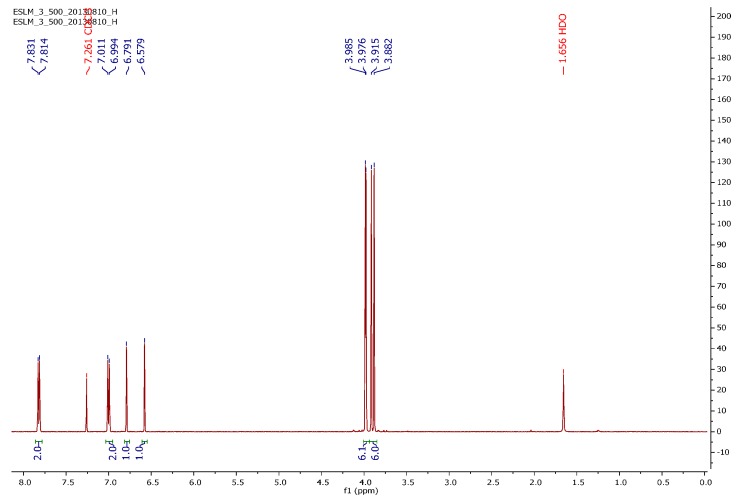
The ^1^H NMR spectrum of TMScu (**1**) observed at 499.88 MHz for CDCl3 solution at 25 °C.

**Figure 8:**
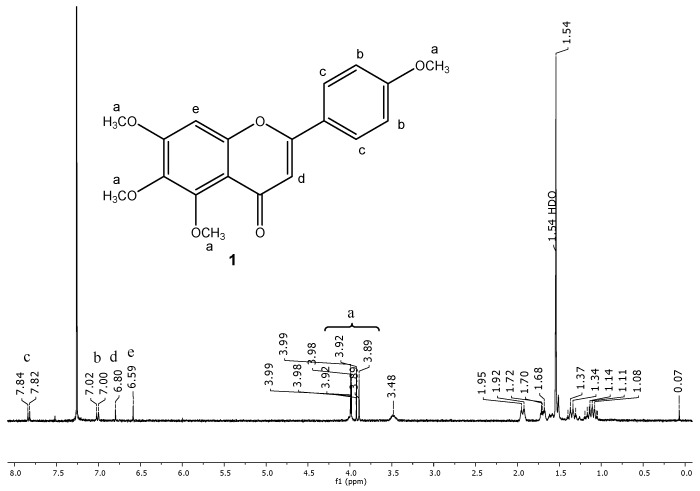
The ^1^H NMR of spectrum of PAMAM G4-TMScu (**1**) complex observed at 400 MHz for CDCl_3_ solution at 25 °C.

**Figure 9:**
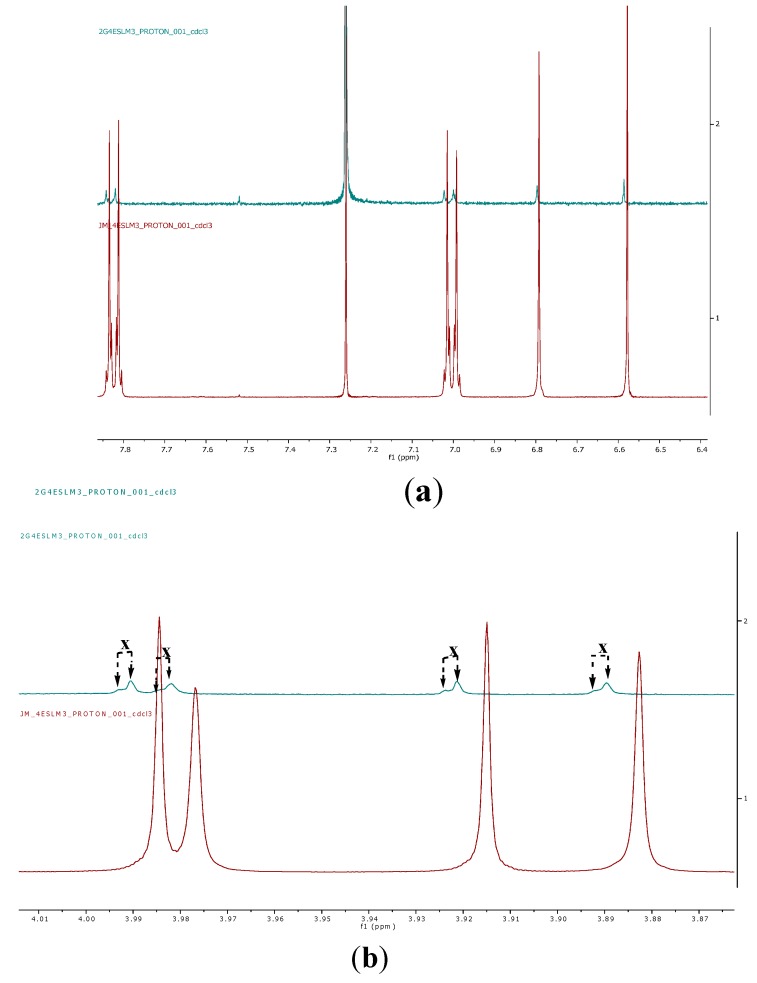
(**a**) An expansion of the ^1^H NMR of spectrum of TMScu (**1**) encapsulated in PAMAM G4 complex (green) stacked with the ^1^H NMR of spectrum of TMScu (**1**) without PAMAM G4 dendrimer observed at 400 MHz for CDCl3 solution at 25 °C; (**b**) An expansion of the ^1^H NMR of spectrum of TMScu (**1**) encapsulated in PAMAM G4 complex (green) stacked with the ^1^H NMR of spectrum of TMScu (**1**) without PAMAM G4 dendrimer observed at 400 MHz for CDCl3 solution at 25 °C (**x** = drifted signals illustration).

The ^1^H NMR spectra of pure compound **1** and its dendrimer complex are presented in Figure 7 and Figure 8, respectively. As indicated in Figure 7, the ^1^H NMR spectrum of the complex (PAMAM G4 TMScu (**1**)) contains signals from both the PAMAM G4 and compound **1**. The appearance of the slight shift of some peaks of compound **1** in the compound–dendrimer complex as illustrated by stacked ^1^H NMR spectra (Figure 9a,b) suggests interactions brought about by an external electrostatic interaction between TMScu (**1**) and PAMAM. The methoxy protons showed significant signal dynamics with approximately δ 0.01 shifts and visually evident drifted signals showing doubling in PAMAM G4–TMScu complex (green, Figure 9b) suggesting interaction probably involving hydrogen bonding between methoxy oxygen of **1** and amino groups of the dendrimer G4.

### 2.3. Encapsulation Efficiency (EE) and Loading Capacity (LC)

Encapsulation efficiency (EE) of compound **1** (C_19_H_18_O_6_, M = 342.3 g/mol) was 77.8% ± 0.69%, while its loading capacity (LC) was 6.2% ± 0.06%. The encapsulation efficiency is influenced by among other factors the nature of the polymer nanoparticle and the encapsulated drug molecules, polymer–drug ratio and the concentration [1,22]. The value obtained for encapsulation efficiency and loading capacity could be attributed to the intrinsic features of both PAMAM G4 dendrimer and TMScu (**1**) enabling intermolecular interactions. Furthermore, dendrimers with high molecular weight such as PAMAM G4 would have high loading capacity and encapsulation capacity as opposed to those with lower molecular weight [22]. The phenomenon is also explained by the results of the present study, the EDTA core G4 dendrimer has high molecular weight as well as larger core size and thus has higher loading capacity as observed.

### 2.4. Phase Solubility Studies of the TMScu (**1**)-Dendrimer G4 Formulation

**Figure 10:**
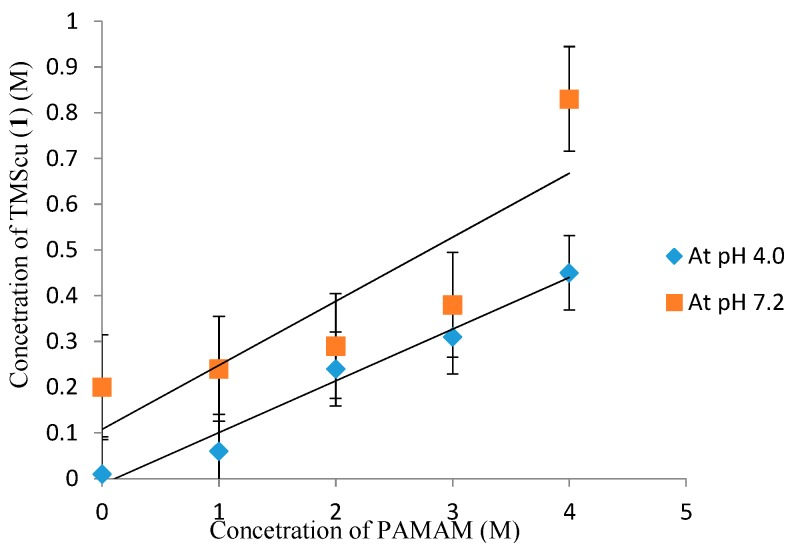
Phase solubility diagram of TMScu (**1**) in the presence of increasing PAMAM concentration.

Measurements of solubilization ability of PAMAM dendrimer TMScu (**1**) was done, as previously described [5]. Phase solubility diagram was obtained by plotting the equilibrium concentration of compound **1** against PAMAM dendrimer concentrations (Figure 10). The solubility increased linearly as function of PAMAM dendrimer concentration albeit with some deviations noticeable at higher concentrations of the dendrimer at pH 7.2. According to the model developed by Higuchi and Connors [23], the linear relationship between TMScu (**1**) solubility and PAMAM dendrimer concentration indicated an A_L_-type phase diagram particularly at pH 4. The obtained linear diagrams (Figure 9) suggest the formation of water-soluble complex in solution due to electrostatic and hydrogen bonding interactions. Such an A_L_-type phase solubility profile is obtained when the complex is first order with respect to both the dendrimer (ligands) and the drug (substrate) [23,24]. Generally, the solubility of drug–dendrimer complex increases linearly with increasing dendrimer concentration due to electrostatic interactions between the interactive functional groups of the drug and the amino groups of the dendrimer [3,25]. The reported mechanism of interaction could hold the same for compound **1**, as it contains the carbonyl and methoxy functionalities in its structure. Thus, the electrostatic interaction between the primary amine of PAMAM G4 and the carboxyl or methoxy groups of the investigated compound could be responsible for its enhanced solubilization. Furthermore, low solubility at pH 4.0 can be attributed to the differences in p*K_a_* of PAMAM dendrimer at different pH levels. The study by Jun-Jun *et al.*, found that the amino groups of the dendrimer have different p*K_a_* [25]. The surface of PAMAM dendrimer amines has p*K_a_* that ranges from 7 to 9 while that of tertiary amines ranges from 3 to 6 [25]. Hence as for the present study, the difference in p*K_a_* of the PAMAM G4 dendrimer would suggest that dissociation would change with variation of pH leading to the variation of solubility with pH.

### 2.5. In Vitro Release of the Loaded TMScu (**1**)

**Figure 11:**
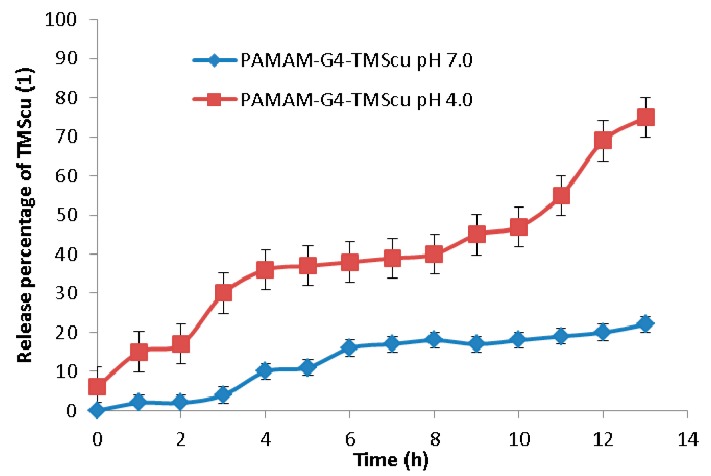
Release profile studies of loaded TMScu (**1**) from dendrimer–compound complex at pH 4 and 7.

*In vitro* release to investigate the stability and interaction of TMScu-dendrimer complex was done in a phosphate buffered saline (PBS) media at two different pH values (4.0 and 7.0). *In vitro* release behavior of loaded compound is presented in Figure 11. For a period of 1 h at pH 4.0, TMScu (**1**) showed an initial release of 17%, which later increased up to 36% for a period of 4 h. The release was nearly maintained for 3 h (*i.e.*, up to the seventh hour) and then progressively increased. The highest release of 75% was obtained after 13 h. However, at pH 7.0, the release of TMScu (**1**) was only 22% for a period of 13 h. As indicated in Figure 11, the *in vitro* release of the investigated compound was pH and ionic interaction dependent. This gives an implication that at pH 7 the TMScu–dendrimer complex formulation was relatively stable as few percentages of the compound were released as compared to that at pH 4. It is generally acknowledged that encapsulation of the compound by amine in basic condition prevents release from the dendrimer [26]. Once the dendrimer enter the cancerous cells or tumor cells which has acidic environment (low pH) the amino groups protonate, repel and undergoes conformational change which facilitate the release of the compounds [26]. More molecules of TMScu (**1**) were released at pH 4.0, implying it could also be made available to the targeted tumor cells’ physiological environment.

### 2.6. Stability Studies of Dendrimer G4-TMScu (**1**) Formulation

The study of the physical stability of the formulations at different temperature conditions, that is, 0, 27 and 40 °C to assess the disintegration was carried out and the results presented in Table 1. For a period of two months, a sign of color changes, turbidity and precipitate formation were observed for formulation stored in light at 40 °C. The formulations/complexes were found to be relatively stable, even at the warmer temperature of 40 °C when stored under dark conditions, indicating that light had an influence on their stability. The formation of precipitates observed may be attributed to the opening of dendritic structures at higher temperatures [5]. There were no noticeable precipitates, turbidity and color changes for the formulations stored in dark and in light at 27 °C, suggesting that the formulations should be stored in the dark, at low temperature or in a cool place for future applications, should it be found useful.

**Table 1:** Stability of PAMAM G4–TMScu (**1**) Formulation.

Formulation	Parameter	Temperature (°C)					
		Light			Dark		
		0	27	40	0	27	40
PAMAM G4-TMScu (**1**)	Color change	−	−	+++	−	−	+
	Turbidity	−	+	+	−	+	++
	Precipitation	−	+	++	−	−	+++

(−) No changes were observed, (+) changes were observed with varied extent.

## 3. Experimental Section

### 3.1. Materials and Reagents

All chemicals and reagents were obtained from commercial sources and used without further purification, unless otherwise stated. Chemicals and reagents included ethylenediaminetetraacetic acid (EDTA) (Rochelle chemicals, Johannesburg, South Africa, (SA), purity: 99.5%), ethylenediamine (EDA) (M & B, London, UK, purity: 98.5%), acetonitrile (Carloerba reagents, Avenue de Valdonne, 13124 Peypin, France, purity: 99.5%), ethyl acetate (Fisher chemicals, Loughborough, Leics, UK, purity: 99.98%), methanol (Fischer chemicals), dicyclohexylcarbodiimide (DCC) (BDH Ltd., London, UK). *Trans*-2-(3-(4-*tert*-butylphenyl)-2-methyl-2-propenylidene)malononitrile (DCTB, 99.0%; Sigma-Aldrich, Stockholm, Sweden) Tetramethylscutellarein (TMScu, **1**) was obtained from the Natural Products Research Laboratory, Chemistry Department, University of Dar es Salaam. Solvents used were analytical reagent (AR) grade.

### 3.2. General Experimental Procedures

Reactions were run at room temperature unless stated otherwise. Purification and separation techniques included filtration, evaporation, and centrifugation. Glassware were washed and dried in an oven. The ^1^H NMR spectra were obtained in CDCl_3_ using Varian MR 400 and Varian VNMR-S 500 at the Department of Chemistry and Molecular Biology, University of Gothenburg, Sweden. The IR spectra were recorded on Platinum ATR alpha Bruker spectrophotometry and UV-Vis spectroscopy measurements using UV-1800PC Spectophotometer (Shenandoah instruments, Inc., Beijing, China) and were performed at the Chemistry Department, University of Dar es Salaam. The autoflex MALDI-TOF-MS analysis was performed on FlexControl Bruker Daltonics, at Polymer Technolgy Laboratory, Chalmers University of Technology, Gothenburg, Sweden. DCTB was used as the matrix for the MALDI-MS acquisition.

### 3.3. Synthesis of Polyamidoamine (PAMAM) Dendrimer Generation 4

**Scheme 1:**
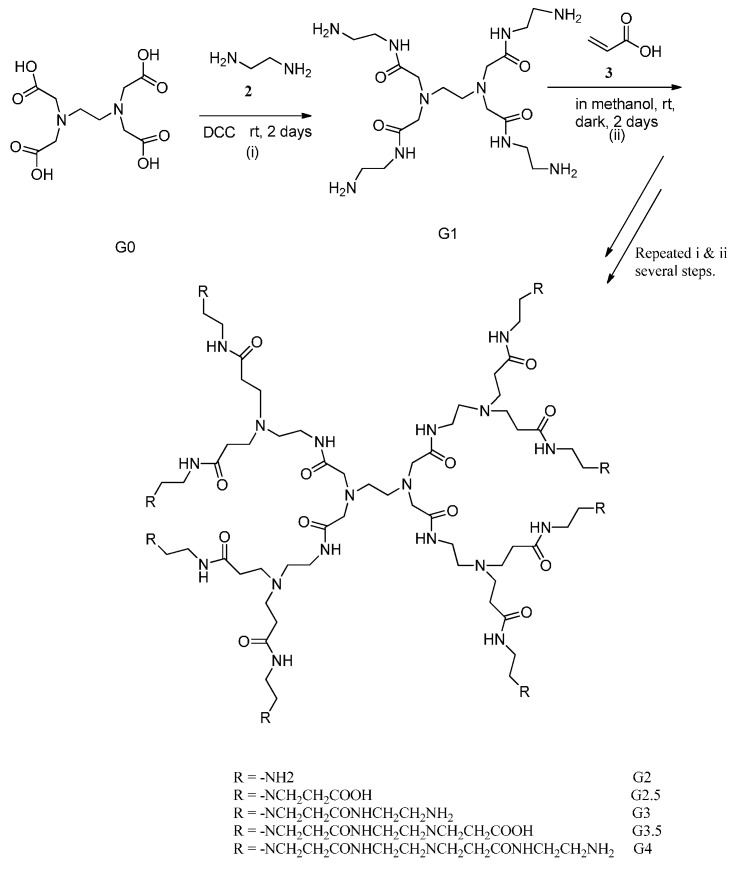
A divergent synthetic route for PAMAM G4 dendrimer.

**Scheme 2:**
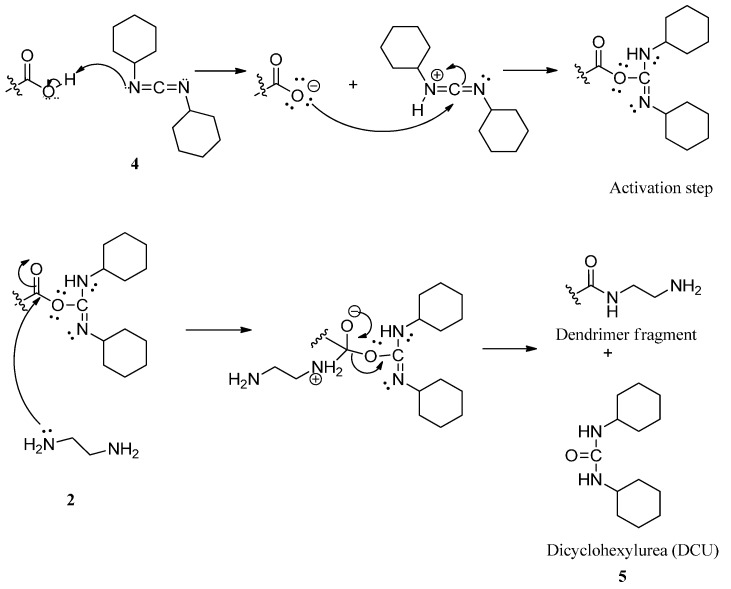
Activation of carboxylic group by Dicyclohexylcarbodiimide (DCC) and amidation step reaction mechanism.

Ethylenediaminetetraacetic acid, EDTA core based PAMAM dendrimers were synthesized by divergent approach with reagent excess using Michael addition and amidation reactions [2]. The Michael addition involved the formation of half dendrimer generations, while in the amidation, two reactions steps were involved: (i) coupling reaction involving activation of carboxylic group and (ii) amidation step, which produced full generation amine terminated G4 PAMAM dendrimer (Scheme 1). In a typical experiment, EDTA core (6 g, 0.0205 mol) in a 250 mL flask was reacted with ethylenediamine (48 mL), EDA (**2**) in the presence of methanol as solvent (100 mL) and dicyclohexylcarbodiimide (4 g) (DCC, **4**) for 48 h in dark while stirring in a tightly stoppered flask. The product obtained G1, was reacted with methacrylic acid (**3**) (30 mL) in the presence of methanol (100 mL) to give G1.5, and the solvent was evaporated by rotary evaporator. Apart from being a coupling and dehydrating agent, DCC was also used to activate the carboxylic group of EDTA. The dicyclohexylurea (DCU, **5**), formed as a byproduct of DCC, was filtered and solvents were evaporated by a rotary evaporator. The activation step reaction was then followed by the amidation reaction (Scheme 2). In this step, the nitrogen lone pair from EDA acted as a nucleophile by reacting with the positive carbonyl carbon from the carboxylic group. These procedures were repeated in several steps to produce the fourth generation dendrimer (PAMAM G4). Synthesized PAMAM G4 was further characterized by IR, UV-Vis, ^1^H NMR spectroscopy and MALDI-TOF-MS.

### 3.4. PAMAM G4

Yellowish oil: UV (λ max) 250 nm; IR (cm^−1^); 3350.71 (N–H stretching of primary amine), 3289.27 and 3182.38 (N–H stretching of secondary amine), 2931.22 and 2861.77 (aliphatic and asymmetric C–H stretching), 1643.79 (stretching of amide carbonyl) and 1565.49 (C–N stretching in a core). ^1^H NMR (CDCl_3_): 4.02 ppm (–CONH–, H_a_); 3.48 ppm (–CONHCH_2_CH_2_NH_2_, H_b_); 1.93 ppm (–CH_2_CONH, H_c_); 1.72 ppm (–CONHCH_2_CH_2_–, H_d_); 1.69 ppm (NCH_2_CH_2_CONH–, H_e_); 1.35 ppm (–NCH_2_CH_2_N–, H_f_ and –N(tertiary)–CH_2_CH_2_, H_f′_).

### 3.5. The Autoflex Matrix-Assisted Laser Desorption/Ionization Time of Flight Mass Spectrum (MALDI-TOF-MS) Analysis

The THF solution of the PAMAM G4 sample and DCTB matrix (1:10 *v*/*v*) was mixed in an Eppendorf tube. Then, 0.5 µL of the resulting solution was deposited on two spots on the stainless microtiter format MALDI target and dried at room temperature to produce a solid layer [15]. The MALDI-TOF mass spectra of PAMAM G4 dendrimer was acquired by 500 laser shots by autoflex when the instrument was operating in the positive ion linear (flight path 1.22 m) mode applying an acceleration voltage of 20.04 kV.

### 3.6. Selection of Model Compound for Encapsulation Study

The selected model compound (TMScu, **1**) with a molecular weight of 342.3 (g·mol^−1^) was a UV active (at 314 nm) and sparingly soluble in aqueous media.

### 3.7. Encapsulation of TMScu (**1**) in PAMAM Dendrimer G4

Encapsulation studies of TMScu, **1** were done according to the reported methods [5,12]. An equal amount of drug to dendrimer ratio in grams was used. At first, 2 mg of compound **1** was dissolved in 10 mL of methanol (*i.e.*, conditions that can dissolve **1**), then 2 mg of the dendrimer was added. The reaction was stirred for 30 h and then the solvent was evaporated under vacuum to remove the methanol completely. This was followed by addition of deionized water to the complex and stirring for another 30 h. Water was filtered and freeze dried to afford a yellow powder of drug–dendrimer complex. Analysis of TMScu (**1**)–PAMAM–G4 complex was done by IR and NMR spectroscopic techniques to determine if there were absorbance changes or chemical shifts of the functional groups of the compound and/or dendrimer in the complex.

### 3.8. Encapsulation Efficiency (EE) and Loading Capacity (LC)

To determine the encapsulation efficiency (EE) of the encapsulated compound **1** in the dendrimer, 2 mg of TMScu (**1**)–PAMAM complex was mixed with 5 mL methanol. The solution was then filtered using membrane of 0.22 µm pore size to exclude encapsulated from non-encapsulated TMScu (**1**) and analyzed by UV-Vis spectrophotometer at absorbance of 314 nm for compound **1**. The encapsulation efficiency was calculated based on the concentration of the encapsulated compound detected in the formulation over the initial concentration of the compound in methanol as per Equation (1) [1].

(1) $$\text{Encapsulation}\;\text{efficiency}\;(\text{EE}\%)\;=\;\frac{\left[W_{t}\right]}{\left[W_{i}\right]}\times 100\%$$

where *W*_t_ is the total amount of compound detected in PAMAM formulation and *W*_i_ is the total quantity of compound added initially during preparation.

The loading capacity (LC) was also calculated based on Equation (2).

(2) $$\text{Loading}\;\text{capacity}\;(\text{LC}\%)\;=\;\frac{\text{Weight}\;\text{of}\;\text{TMScu}\;\text{in}\;\text{PAMAM-G4}}{\text{Weight}\;\text{of}\;\text{PAMAM-G4}}\times 100\%$$

### 3.9. Solubilization Study of TMScu (**1**)

Phase solubility studies of loaded compound (TMScu, **1**) were done according to the previously reported method [5]. The studies were performed in pH of 4.0 and 7.2 to investigate the effect of pH on the solubility of the encapsulated compound. The pH 4.0 was chosen to reflect that of cancerous cells, while pH 7.2 is that of blood at normal physiological conditions. In the experiment, an excess of compound **1** was added into glass vials containing different concentration of PAMAM dendrimers (*v*/*v* 0.1%–0.4%) in pH 7.2 while in another vial containing only water, excess amount of the compound was added and treated as a control. Both vials were shaken at room temperature for 36 h in a shaker (Digital Grant Bio Shaker HY-5A, POS 300, USA) and allowed to stand attain equilibrium. The undissolved compound was removed by filtration through a 0.22 µm filter paper membrane size and washed twice with distilled water then solubilized in methanol and analyzed by UV-Vis spectrophotometer at the compound’s absorbance wavelength. A similar procedure was repeated at pH 4.0 for triplicate experiments and the mean values were used to plot the graphs.

### 3.10. In Vitro Release Studies of Loaded Compound (TMScu, **1**)

*In vitro* release studies of the loaded compound (TMScu, **1**) from PAMAM dendrimer–TMScu complex were measured in triplicate in a PBS media at pH 7.0 and pH 4.0. One milligram of dendrimer–compound complexes was suspended in 5 mL of buffer solution (pH = 7.0 and 4.0) in a screw-capped vial. The vials were then placed in a shaker maintained at 37 °C and shaken horizontally at 240 rpm at a predetermined time interval. The vials were then removed from the shaker and centrifuged for 10 min. The supernatant collected from the vials were then subjected for UV-Vis analyses. The precipitated dendrimer were re-suspended in 5 mL of fresh buffer solution and then put back into the shaker for continuous *in vitro* release measurements as per previously described method [21].

### 3.11. Stability Studies of TMScu (**1**)

The principles of chemical kinetics were employed in the stability studies of the formulation [5]. A stability study was therefore carried out under light and dark conditions with different temperature settings. In the stability study, 1 mL of formulations was kept in dark (using amber-colored glass vials) and in light (colorless vials) at 0, 27 °C (room temperature) and 40 °C in a controlled oven for 2 months. Samples were periodically withdrawn and analyzed for any color changes, crystallization, precipitation and turbidity.

## 4. Conclusions

This study investigated the encapsulation and solubilization capacity of the as-synthesized PAMAM dendrimer G4 on a flavonoid tetramethylscutellarein (TMScu, **1**) as an approach to enhance the potency of the flavonoid molecules. The synthesis of EDTA core based PAMAM dendrimer of generation four (G4) was designed and successfully achieved via a divergent approach by reacting EDA and acrylic acid in repeated steps. The synthesized G4 dendrimer was characterized by UV-Vis, IR and NMR spectroscopy. The encapsulation of the studied flavonoid (TMScu, **1**) into PAMAM G4 dendrimer, solubilization and *in vitro* release were explored using a combination of IR, UV-Vis and NMR spectroscopic techniques. It was established that amine-terminated dendrimer G4 formed a complex with the flavonoid due to hydrogen bonding and electrostatic interaction as observed in the IR and NMR signal absorption changes. The amine terminated PAMAM G4 enhanced the solubility of the studied compound as a function of dendrimer concentration. Furthermore, pH was established to have an influence on the solubility with solubility being higher at pH 4 than at pH 7. The *in vitro* release studies indicated that PAMAM G4-MScu complex was relatively more stable in neutral medium at pH 7 than at pH 4. Overall, it has been established that PAMAM G4 dendrimer is capable of encapsulating TMScu and hence enhancing its solubility. The dendrimer thus has potential for the bioactivity enhancement of the studied and other related compounds warranting further investigation under *in vivo* and or *in vitro* bioassay setups.
